# Adolescent obesity and ANGPTL8: correlations with high sensitivity C-reactive protein, leptin, and chemerin

**DOI:** 10.3389/fendo.2023.1314211

**Published:** 2023-12-22

**Authors:** Maha M. Hammad, Arshad M. Channanath, Mohamed Abu-Farha, Abdur Rahman, Irina Al Khairi, Preethi Cherian, Tahani Alramah, Nada Alam-Eldin, Fahd Al-Mulla, Thangavel Alphonse Thanaraj, Jehad Abubaker

**Affiliations:** ^1^ Department of Pharmacology and Toxicology, Faculty of Medicine, Kuwait University, Kuwait City, Kuwait; ^2^ Genetics and Bioinformatics Department, Dasman Diabetes Institute, Kuwait City, Kuwait; ^3^ Biochemistry and Molecular Biology Department, Dasman Diabetes Institute, Kuwait City, Kuwait; ^4^ Department of Food Science and Nutrition, College of Life Sciences, Kuwait University, Kuwait City, Kuwait

**Keywords:** adolescents, angiopoietin-like protein 8, chemerin, high sensitivity C-reactive protein, leptin, obesity, Arab ethnicity

## Abstract

Angiopoietin-like proteins (ANGPTLs) mediate many metabolic functions. We had recently reported increased plasma levels of ANGPTL8 in obese adults of Arab ethnicity. However, data on ANGPTL8 levels in adolescent obesity is lacking. Arab population is characterized by a rapid transition, due to sudden wealth seen in the post-oil era, in lifestyle, food habits and extent of physical activity. We adopted a cross-sectional study on Arab adolescents from Kuwait to examine the role of ANGPTL8 in adolescent obesity. The study cohort included 452 adolescents, aged 11-14 years, recruited from Middle Schools across Kuwait. BMI-for-age growth charts were used to categorize adolescents as normal-weight, overweight, and obese. ELISA and bead-based multiplexing assays were used to measure plasma levels of ANGPTL8 and other inflammation and obesity-related biomarkers. Data analysis showed significant differences in the plasma levels of ANGPTL8 among the three subgroups, with a significant increase in overweight and obese children compared to normal-weight children. This observation persisted even when the analysis was stratified by sex. Multinomial logistic regression analysis illustrated that adolescents with higher levels of ANGPTL8 were 7 times more likely to become obese and twice as likely to be overweight. ANGPTL8 levels were correlated with those of hsCRP, leptin and chemerin. ANGPTL8 level had a reasonable prognostic power for obesity with an AUC of 0.703 (95%-CI=0.648-0.759). These observations relating to increased ANGPTL8 levels corresponding to increased BMI-for-age z-scores indicate that ANGPTL8, along with hsCRP, leptin and chemerin, could play a role in the early stages of obesity development in children. ANGPTL8 is a potential early marker for adolescent obesity and is associated with well-known obesity and inflammatory markers.

## 1 Introduction

Childhood obesity is a major public health concern worldwide. As per the World Health Organization (WHO) obesity report for 2023, the global prevalence of overweight or obesity among children and adolescents (5–19 years of age) has quadrupled, from 4% in 1975 to 18% in 2016 (1). An extremely high prevalence of childhood obesity is observed in the Gulf region of the Arabian Peninsula (2). In Kuwait, the latest statistics indicate that the prevalence of overweight or obesity among adolescents reached nearly 50% with 54% in males and 45% in females (3). Several factors contribute to this alarmingly high prevalence rate of obesity among adolescents, including an indoor lifestyle, lack of physical activity, and extensive availability of high-calorie fast foods. The Arabian Gulf countries witnessed in the post-oil rich era a rapid urbanization in everyday life, accompanied by decreased levels of physical activity and increased caloric intake of non-traditional food – the rapid transition has become responsible for the emerging of obesity in children and adolescents as a major public health issue in these countries. The Arabian Gulf nations are a good example for this rapid developmental transition and its consequences. Childhood and adolescent obesity often persist into adulthood and leads to several chronic diseases, including diabetes, cardiovascular disease, and cancer, affecting the quality of life. Obesity in childhood and adolescence can lead to diabetes forms such as early-onset type 2 diabetes. Thus, addressing this epidemic of obesity needs urgent attention. The key to a successful approach to curtail obesity epidemic lies in early detection and prevention. As part of approaches for early detection, identifying potential biomarkers in diagnosing and progressing these conditions is crucial. To develop therapeutic strategies to address these problems, it is important to explore the molecular pathways that contribute to the development of obesity and metabolic syndrome.

Angiopoietin-like protein 8 (ANGPTL8), also known as betatrophin, is a member of the angiopoietin-like proteins family. The family consists of eight members (ANGPTL1 through ANGPTL8) and is involved in regulating several physiological functions in lipid and glucose metabolism, inflammation, hematopoiesis, angiogenesis, and cancer (4–6). The important role of the ANGPTL family in the regulation of a plethora of physiological and pathophysiological processes has prompted researchers to assess the circulatory levels of ANGPTLs in various disease conditions in different age groups and ethnicities. Studies from our group as well as other investigators reported significant differences and associations between certain ANGPTL proteins and obesity, hypertension, insulin resistance, glucose tolerance, and inflammation (7–13). For example, we have shown that levels of ANGPTL8 were significantly elevated in adult individuals afflicted with obesity, diabetes, and metabolic syndrome compared with healthy control individuals (10–12). Some studies also focused on the ANGPTLs and their associations with metabolic diseases in children and adolescents. To cite an example, we reported a correlation between higher circulating ANGPTL5 levels and obesity, high sensitivity C-reactive proteins (hsCRP), and oxidized low-density lipoprotein (ox-LDL) in adolescents (14). Having seen a positive correlation between ANGPTL8 levels and adult obesity, and positive correlations between ANGPTL5 and obesity levels in Arab children, we were motivated to examine the ANGPTL8 levels in the context of adolescent obesity. A prospective cohort study assessed the serum levels of both ANGPTL3 and ANGPTL8 in Korean children (15) in the context of obesity and lipid profiles. The Arab population differs from other ethnic populations in terms of the rapid transition in lifestyle brought by the post-oil wealth. The sudden developmental transition is unique to the Arab population. Thus, characterizing the different members of the ANGPTL family in such a population is of interest.

In this study, we aimed to assess the circulating levels of ANGPTL8 in adolescents with overweight or obesity in comparison to its levels in normal-weight adolescents; and to investigate its associations with the levels of some established obesity markers. We hypothesize that, like our earlier observations in adults, plasma ANGPTL8 levels are positively associated with obesity and hsCRP in adolescents.

## 2 Materials and methods

### 2.1 Study participants

This cross-sectional study included adolescents recruited from public middle schools in the State of Kuwait as previously described (16, 17). Students with major chronic disease conditions were excluded from the study. A total of 425 randomly selected participants from the original cohort were included in the present study; the size of 425 optimizes the cost for measuring plasma levels of biomarkers; the size is expected to have enough power for deriving meaningful statistical observations. The participants were in the age group of 11 to 14 years. None of the 425 participants was diagnosed with any type of diabetes.

### 2.2 Ethics, consent, and permissions

The study was approved by The Ethics Committee of the Ministry of Health, Kuwait (No: 2015/248), The Ethics Committee of the Health Sciences Centre, Kuwait University (No: DR/EC/2338), and the Ethical Review Committee of Dasman Diabetes Institute (RA2017-026). Parents of the adolescent participants signed the informed written consent form prior to the enrolment of participants in the study. Verbal assent of every study participant was also obtained.

### 2.3 Blood collection

5mL of venous blood was collected in gel-containing tubes (SST II Advance, BD Vacutainer) from each participant. The collected blood sample was centrifuged to separate the plasma. Plasma was aliquoted and stored at −80°C.

### 2.4 Anthropometric measurements

A digital weight and height scale (Detecto, Webb City, MO, USA) was utilized to measure the body weight and standing height of each study participant while the participant was standing erect barefooted and wearing light clothes.

### 2.5 ELISA measurements for the levels of ANGPTL8 and hsCRP

Plasma samples were used to measure the levels of biomarkers. ANGPTL8 levels were determined using ELISA kit (Cat. # 30145444, IBL, Gunma, Japan) with optimal dilution of 1:20. hsCRP concentrations were determined using ELISA kit (Cat. # HK369; Hycult Biotech) with optimal dilution of 1:1000. Assays were performed following the manufacturer’s instructions.

### 2.6 Multiplexing assay for the levels of leptin, chemerin, and other obesity markers

Bead-based multiplexing technology on a Bio-Plex 200 system (Bio-Rad) was used to determine the levels of several obesity and inflammatory markers. We used a custom-made 12-plex kit for the leptin, chemerin, IL-6, IL-10, and visfatin and performed the assays according to the manufacturer’s protocol.

### 2.7 Statistical analysis

BMI-for-age z-scores were calculated using WHO growth charts. Participant was considered obese when BMI-for-age was ≥+3 Standard Deviation (SD), overweight when BMI-for-age was >+2 SD and <+3 SD, and normal-weight otherwise. Matching of individuals in the three subgroups was assessed by evaluating the statistical significances of differences in age, sex, and school year (class in the school). All measurements were reported as mean (SD). Correlations between the levels of ANGPTL8 and those of hsCRP, leptin, chemerin, and IL-10 were depicted graphically, and Spearman correlation coefficients were calculated. Continuous values of plasma levels for the biomarkers were categorized into tertiles in order to examine the relationships between these biomarkers and the risk of being overweight or obese. Multinomial logistic regression was conducted to explore the relationship between BMI levels (categorical dependent variable: normal, overweight, or obese) and tertiles of biomarkers (categorical independent variable: lower tertile, middle tertile, and higher tertile). The lower tertile group was chosen as the reference category for comparison with the other tertile groups (middle tertile and higher tertile). The reference tertile serves as a baseline for assessing the relative odds of being overweight or obese across the different tertile groups of the biomarkers. Pearson’s Chi-squared test, Fisher’s exact test, and Kruskal-Wallis rank sum test were used to determine the differences, and those with p<0.05 were considered statistically significant. Receiver Operating Characteristic (ROC) analysis was conducted to evaluate the predictive performance of ANGPTL8 for obesity. Individuals having missing data for a particular biomarker were not included in the analysis involving the biomarker.

## 3 Results

### 3.1 Characteristics of the study participants based on their BMI-for-age classification

This study used a sub-cohort of a large cohort that was described in our previous studies (14, 16). A total of 425 participants were included in the current study. Participants were grouped, using BMI-for-age growth charts, into three study subgroups, namely normal-weight (N=193), overweight (N=89), and obese (N=143). Clinical characteristics of the cohort in terms of these three study groups are presented in **Table 1**. Differences in age, sex, and school year (class in the school) among the three subgroups were statistically insignificant indicating that the subgroups are matched. Levels of ANGPTL8, hsCRP, leptin, IL-6, and chemerin were significantly different (p<0.001) among the three subgroups and level of IL-10 was different among the subgroups at borderline p-value of 0.045.

**Table 1:** Summary characteristics of study participants categorized by obesity status based on BMI-for-age growth charts.

Characteristic feature	Normal-weight N = 193^1^	Overweight N = 89^1^	Obese N = 143^1^	p-value^2^
Sex				
Female	107 (55%)	49 (55%)	76 (53%)	>0.9
Male	86 (45%)	40 (45%)	67 (47%)	
**Age (years)**	12.32 (0.87)	12.33 (0.81)	12.30 (0.85)	0.9
Grade (class in the middle school)				
6	94 (49%)	37 (42%)	69 (48%)	0.6
7	68 (35%)	31 (35%)	47 (33%)	
8	31 (16%)	21 (24%)	27 (19%)	
Age category				
10 - <12 years	82 (42%)	37 (42%)	62 (43%)	0.8
12 - <13 years	67 (35%)	28 (31%)	52 (36%)	
13+ years	44 (23%)	24 (27%)	29 (20%)	
**Glucose (mmol/l) - Mean (SD)**	4.87 (0.72)	4.87 (0.74)	5.22 (1.76)	0.11
**hsCRP (µg/ml) - Mean (SD)**	0.63 (0.99)	1.45 (2.06)	3.34 (2.60)	<0.001
**ANGPTL8 (pmol/l) - Mean (SD)**	110 (52)	127 (58)	150 (58)	<0.001
**IL-10 (pg/ml) - Mean (SD)**	1.77 (1.93)	1.34 (1.70)	2.06 (7.35)	0.045
**Leptin (ng/ml) - Mean (SD)**	55.1 (47.6)	125.8 (71.5)	255.8 (141.7)	<0.001
**IL-6 (pg/ml) - Mean (SD)**	0.25 (0.80)	0.50 (2.15)	1.09 (7.25)	<0.001
**TNF-alpha (pg/ml) - Mean (SD)**	0.78 (1.39)	0.68 (1.79)	1.75 (11.24)	0.3
**Visfatin (pg/ml) - Mean (SD)**	4,360 (2,777)	4,223 (2,693)	5,172 (2,775)	0.14
**Chemerin (ng/ml) - Mean (SD)**	4.55 (1.66)	5.33 (2.47)	5.51 (2.25)	<0.001

^1^n (%); Mean (SD).

^2^Pearson’s Chi-squared test; Fisher’s exact test; Kruskal-Wallis rank sum test.

### 3.2 Plasma ANGPTL8 levels are significantly elevated in adolescents with overweight and obesity

**Figure 1:**
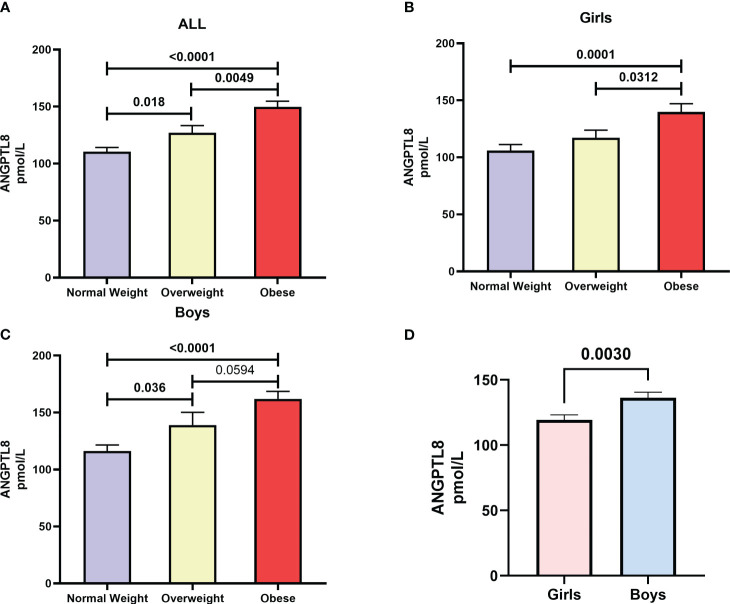
Distribution of Angiopoietin-like protein 8 (ANGPTL8) in **(A)** adolescents who are normal-weight, overweight, and obese from the study cohort, **(B)** female adolescents who are normal-weight, overweight, and obese, **(C)** male adolescents who are normal-weight, overweight, and obese, **(D)** girls versus boys irrespective of their obesity status.

We found a significant increase (p < 0.05) in ANGPTL8 levels as subgroups of increasing BMI-for-age z-scores, namely normal-weight, overweight, and obese, were considered. The mean (SD) levels of ANGPTL8 were 110.4 (52.1) pmol/L, 127.1 (57.94) pmol/L, and 149.7 (57.99) pmol/L in normal-weight, overweight and obese, respectively (**Figure 1A**). The difference in ANGPTL8 levels between the obese subgroup and the overweight or normal-weight were at p-values of 0.005 and <0.0001, respectively. When we stratified the analysis by sex, we still observed an elevated level of ANGPTL8 in female and male individuals with obesity compared to normal-weight individuals (**Figures 1B, C**). In addition, as shown in **Figure 1D**, ANGPTL8 levels were significantly lower (p=0.003) in girls [119.4 (57.4) pmol/L] than in boys [136.4 (57.2) pmol/L]. Multinomial logistic regression analysis showed that the odds of being obese, and overweight were significantly associated with ANGPTL8 levels (**Table 2**). The odds ratio [OR (95% CI)] for individuals characterized with upper tertile of ANGPTL8 level was 1.95 (1.04-3.66) for being in the overweight group and 7.08 (3.81-13.1) for being in the obesity group.

**Table 2:** Results of multinomial regression analysis reporting odds ratios for the risk of being overweight or obese due to levels of biomarkers^@^.

Biomarker	Overweight			Obese		
	OR^1^	95% CI^1^	p-value	OR^1^	95% CI^1^	p-value
ANGPTL8 – tertile categories (and category boundaries in pmol/l).						
Lower tertile (11.9 – 93.5)	Reference category. OR=1			Reference category. OR=1		
Middle tertile (93.5 – 143.1)	1.03	0.56- 1.90	>0.9	2.73	1.48- 5.02	0.001
Higher tertile (143.1 – 359.8)	1.95	1.04- 3.66	0.037	7.08	3.81- 13.1	<0.001
hsCRP – tertile categories (and category boundaries in µg/ml).						
Lower tertile (0.0 – 0.29)	Reference category. OR=1			Reference category. OR=1		
Middle tertile (0.29 – 1.58)	4.74	2.51- 8.93	<0.001	7.34	3.17- 17.0	<0.001
Higher tertile (1.58 – 9.90)	5.25	2.43- 11.3	<0.001	49.5	21.2- 116	<0.001
Leptin tertile categories (and category boundaries in ng/ml).						
Lower tertile (1.80 – 55.80)	Reference category. OR=1			Reference category. OR=1		
Middle tertile (55.80 – 153.10)	5.42	2.80- 10.5	<0.001	13.8	4.67- 41.0	<0.001
Higher tertile (153.10 – 548.70)	34.3	12.8- 91.6	<0.001	437	124- 1,536	<0.001
Chemerin tertile categories (and category boundaries in ng/ml).						
Lower tertile (0.34 – 4.20)	Reference category. OR=1			Reference category. OR=1		
Middle tertile (4.20 – 5.65)	1.00	0.54- 1.84	>0.9	1.48	0.84- 2.62	0.2
Higher tertile (5.65 – 11.24)	1.98	1.05- 3.75	0.035	4.85	2.74- 8.61	<0.001

^1^OR, Odds Ratio; CI,Confidence Interval.

**^@^,**The continuous values of plasma levels for the biomarkers were categorized into tertiles to examine relationships between these biomarkers and the risk of being overweight or obese. Obesity levels were categorized into three types, namely normal-weight, overweight and obese, based on BMI-for-age growth charts. The participant was considered obese when BMI-for-age was ≥+3 Standard Deviation (SD), overweight when BMI-for-age was >+2 SD and <+3 SD, and normal-weight otherwise. The category boundaries for tertiles of the biomarkers are presented in column 1.

### 3.3 Children with obesity have significantly higher levels of hsCRP, leptin and chemerin

**Figure 2:**
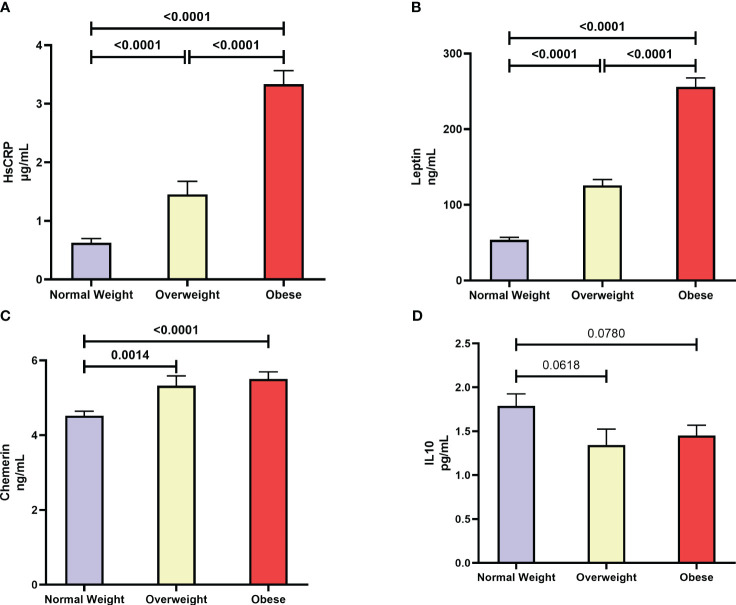
Distribution of **(A)** high sensitivity C-reactive protein (hsCRP), **(B)** Leptin, **(C)** Chemerin, and **(D)** IL-10 in subgroups of normal-weight, overweight, and obese individuals.

The mean (SD) levels of hsCRP were 0.63 (0.99) µg/mL, 1.45 (2.06) µg/mL, and 3.34 (2.6) µg/mL in the subgroups of normal-weight, overweight, and obese individuals, respectively (**Figure 2A**). *Post hoc* analysis showed significant differences in hsCRP levels in each of the overweight vs. normal-weight, obese vs. overweight, and obese vs. normal-weight comparisons [p < 0.0001]). Leptin levels also displayed the same pattern as hsCRP showing increased levels with obesity. The mean (SD) levels of leptin were 55.1 (47.6) ng/mL, 125.8 (71.54) ng/mL, and 255.8 (141.7) ng/mL in normal-weight, overweight, and obese, respectively (**Figure 2B**). *Post hoc* analysis showed significant differences in leptin levels between normal-weight vs. overweight, overweight vs. obese, and obese vs. normal-weight comparisons [p < 0.0001]). In addition, when chemerin levels were assessed, there was a significant elevation (p ≤ 0.0014) in overweight 5.33 (2.47) ng/mL and obese 5.51 (2.25) ng/mL groups compared to normal-weight group 4.55 (1.66) ng/mL (**Figure 2C**). We also evaluated the levels of IL-10 in our cohort and observed a trend for reduction with increased obesity - mean (SD) values of IL-10 were 1.79 (1.92) pg/mL, 1.34 (1.7) pg/mL, and 1.45 (1.4) pg/mL in normal-weight, overweight and obese groups, respectively, *albeit* with no statistical significance (**Figure 2D**).

### 3.4 ANGPTL8 level correlates with levels of hsCRP, leptin and chemerin

**Figure 3:**
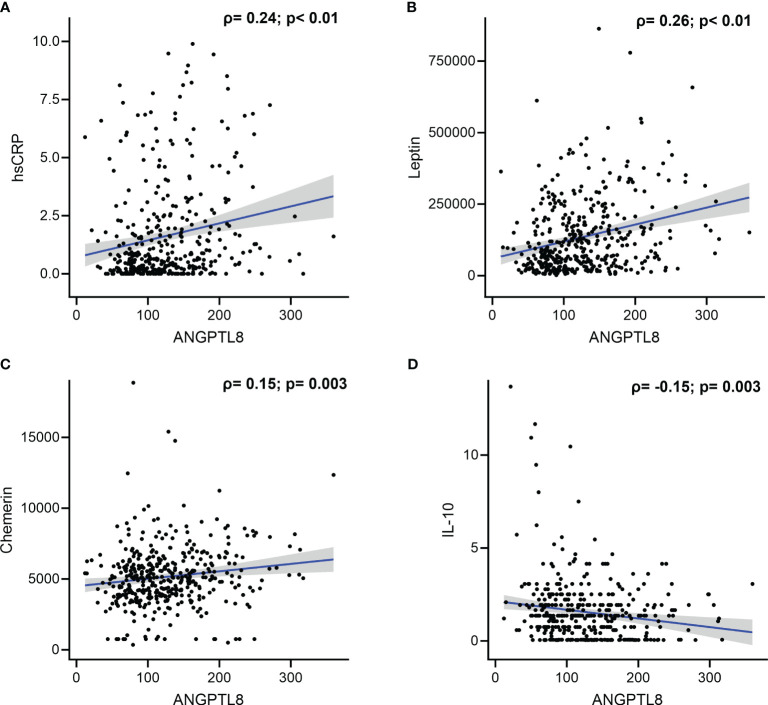
Correlation between Angiopoietin-like protein 8 (ANGPTL8) and selected markers: **(A)** high sensitivity C-reactive protein (hsCRP), **(B)** Leptin, **(C)** Chemerin, and **(D)** IL-10.

We found a positive correlation between ANGPTL8 and hsCRP levels (Spearman’s correlation coefficient, ρ = 0.24, p < 0.001; **Table 3**, **Figure 3A**). Circulating ANGPTL8 levels were also positively correlated with leptin levels (Spearman’s correlation coefficient, ρ = 0.26, p < 0.001; **Table 3**, **Figure 3B**). Similarly, we observed a positive correlation between the levels of ANGPTL8 and chemerin (ρ = 0.15, p = 0.003; **Table 3**, **Figure 3C**). On the other hand, there was a negative correlation between ANGPTL8 and IL-10 levels (ρ = -0.15, p = 0.003; **Table 3**, **Figure 3D**).

**Table 3:** Correlations between the levels of ANGPTL8 and biomarkers.

Biomarker	Correlation coefficient (ρ)^@^	p-value
hsCRP	0.24	<0.001
Leptin	0.26	<0.001
Chemerin	0.15	0.003
IL-10	-0.15	0.003
IL-6	0.061	0.215
Visfatin	0.051	0.503

**^@^**, Spearman correlation coefficient.

### 3.5 Receiver operating characteristic curve analysis of ANGPTL8

**Figure 4:**
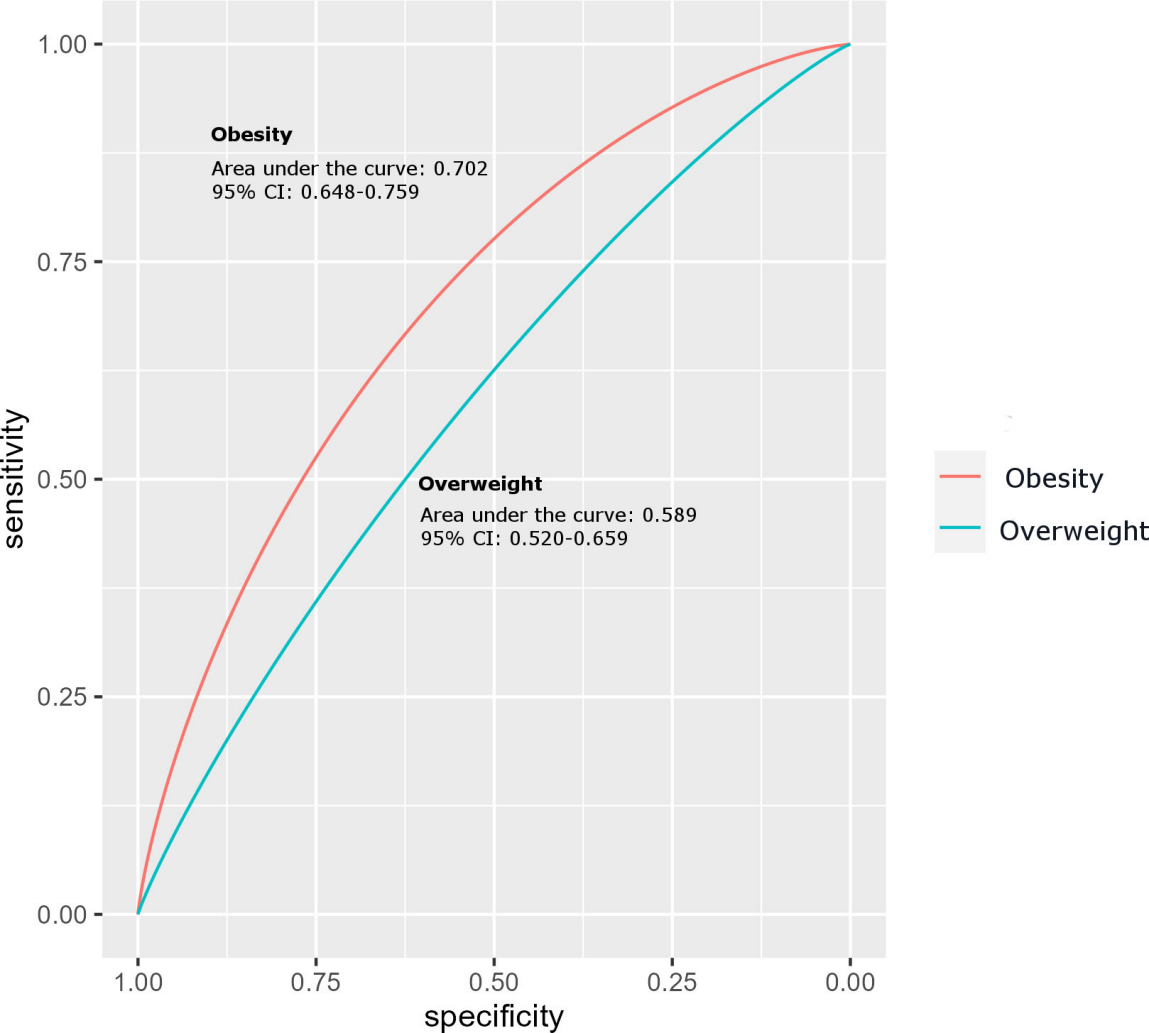
Receiver Operating Characteristic (ROC) curve analysis for ANGPTL8 of obese group compared to the non-obese control group of individuals.

Results of ROC analysis to evaluate the predictive performance of ANGPTL8 on obesity showed that ANGPTL8 has reasonable predictive power, with an area under curve (AUC) of 0.703 (95% CI 0.648 - 0.759) indicating acceptable accuracy in predicting obesity (**Figure 4**). The optimal threshold of ANGPTL8 level for distinguishing individuals with obesity from those with no obesity (i.e., both the normal-weight and overweight groups from the category of non-obese individuals) was found to be 122.8 pmol/l. At this threshold, the sensitivity was 0.67 (i.e., ANGPTL8 correctly identified 67% of individuals with obesity as true positives) and the specificity was 0.64 (i.e., correctly calling 64% of non-obese individuals as true negatives). However, ANGPTL8 was less effective in distinguishing overweight individuals from normal-weight individuals – the observed AUC was 0.589 (95% CI 0.520-0.659).

## 4 Discussion

Childhood obesity, which is reaching epidemic levels, is a major public health concern given its contributory role in the development of other metabolic disorders such as diabetes and cardiovascular diseases. Thus, it is important to identify biomarkers that are predictive of obesity. ANGPTLs have been shown to play crucial roles in metabolic pathways relating to lipid and glucose metabolism as well as to inflammation – particularly, the elevated levels of ANGPTL proteins have been linked to the pathogenesis of obesity, diabetes, and metabolic syndrome (7, 9, 11–13). Our group published several articles in the last few years describing the levels of different members of the ANGPTL family in the Kuwaiti Arab population in different types of cohorts (adults, adolescents, adults with hypertension, and adults with diabetes) and delineating the association of ANGPTLs with multiple biomarkers. For example, we showed that ANGPTL8 is strongly associated with hsCRP in adults (10). We later demonstrated increased circulating levels of ANGPTL8 and ANGPTL4 in adults with hypertension (18). We also reported higher ANGPTL5 levels in adolescents with obesity compared to adolescents without obesity (14) and in adults with and without diabetes (19).

In the current study, we investigated the role of ANGPTL8 in childhood obesity and its association with inflammation and metabolic markers, such as hsCRP, leptin, and chemerin. The data presented in the current study showed significant differences in the plasma levels of ANGPTL8 among the three groups (normal-weight, overweight, and obese). A significant increase in plasma ANGPTL8 levels in overweight and obese children compared to normal-weight children was observed. This observation was further confirmed by multinomial logistic regression analysis, which illustrated that adolescents with higher plasma levels of ANGPTL8 were 7 times more likely to become obese and twice as likely to be overweight. Even when the analysis was stratified by sex, statistically significant elevated levels of ANGPTL8 were observed in female and male individuals with obesity compared to normal-weight individuals. These observations relating to increased ANGPTL8 levels corresponding to increased BMI-for-age z-scores indicate that ANGPTL8 could play a role in the early stages of obesity development in children. Interestingly, ANGPTL8 showed an early and significant elevation in the overweight group, unlike our previous data with ANGTPL5 where levels were elevated only in obese adolescents (14). Our findings are not consistent with the few studies that investigated some of the ANGPTL family members in children. Specifically, a study on Korean children in 2015 reported no differences in the levels of either ANGPTL3 or ANGPTL8 between normal-weight children and obese children (15). Another study on Turkish children in 2018 could not also demonstrate significant differences in ANGPTL8 levels between obese and non-obese groups (20). An earlier study performed in China reported no differences in circulating ANGPTL8 levels between lean children and overweight or obese children (21). Tuhan et al., by way of considering a small-sized cohort (20 obese children with insulin resistance, 20 obese children without insulin resistance and 35 non-obese children from Turkey) found that (a) insulin resistant obese subjects had lower serum ANGPTL8 concentrations than non-insulin resistant obese subjects; and (b) circulating ANGPTL8 concentration was negatively correlated with insulin resistance in obese children and adolescents; they further suggested that ANGPTL8 might act as a potential biomarker for insulin resistance in obese children or adolescents (22). It is important to point out that we differ from the above-mentioned reports in the study design. While we distributed our study cohort individuals into three groups (normal-weight, overweight, and obese), the above-cited previous studies combined the overweight and obese participants into one group and thereby might have missed to observe differences in ANGPTL8 levels. Another obvious difference between the previous studies and ours is the ethnicity of the study population. Our study subjects were Arabs, but the previous studies were conducted on Chinese (Asian), Korean (East Asian), and Turkish subjects. Similar conflicting observations across different ethnicities were made in studies on adults with obesity and diabetes (23–26). Further studies on multiple populations are needed to confirm the role of ethnicity in the association between ANGPTL8 and obesity.

Our study showed a significant reduction in ANGPTL8 levels in girls compared to boys. Interestingly, both the previous studies from Korea and China also reported similar observations in their cohorts (15, 21). The sex-specific concentration of ANGPTL8 is probably attributable to differences in developmental stages during adolescence that are accompanied by changes in sex hormones. Considering that sex hormones are associated with body fat composition and adiposity (27), it is appropriate to anticipate that ANGPTL8, which plays an important role in lipid metabolism, would be affected by these changes in sex hormones and in body fat composition during puberty (28). Specifically, female children gain more fat mass during puberty, whereas male children gain more skeletal mass (28, 29). Further, adipokines, such as chemerin, leptin, adiponectin, visfatin, and nesfatin-1, are associated with not only processes pertaining to metabolism but also with processes that coordinate reproductive activities (30, 31). Thus, it is important to consider sex hormone levels as confounders in the analysis of obesity-related marker levels. Future studies measuring the levels of sex hormones and body fat compositions along with ANGPTL8 levels in adolescents are required to validate the observed relationships that ANGPTL8, chemerin, leptin, and CRP have with obesity.

It is now common to define obesity as a low-grade inflammation condition characterized by an elevation of various inflammatory markers (32, 33). The data presented in the current study (see **Table 1**) showed significant increases in the plasma levels of hsCRP across the three subgroups groups of increasing BMI-for-age z-scores (normal-weight, overweight, and obese). Our multinomial regression analysis showed that children with higher levels of hsCRP (upper tertile) were 5 times more likely to be overweight and about 50 times more likely to become obese (see **Table 2**). It is further the case that the levels of hsCRP and ANGPTL8 were correlated (see **Figure 3**). It is known that hsCRP, which is produced in the liver in response to inflammatory signals and cytokines, is a strong marker for detecting low inflammatory processes. Based on previous studies from the literature, elevated CRP levels have been associated with cardiovascular mortality in adults as well as with an increased risk of diabetes and insulin resistance in adults, adolescents, and children (34, 35). Mechanistically, the increase in circulating hsCRP levels is thought to be attributable to the infiltration of the expanded adipose tissue by macrophages, which are responsible for the generation of inflammatory signals and the production of cytokines such as IL-6. It is interesting to note that significant increases in the plasma levels of IL-6 were seen across the three subgroups groups of increasing BMI-for-age z-scores (see **Table 1**). Further, ANGPTL8 level inversely correlated with IL-10, a potent anti-inflammatory cytokine that plays a crucial, and often essential role in preventing inflammatory and autoimmune pathologies (36). Increased production of inflammatory cytokines can therefore result in the production of hsCRP by hepatocytes (37). Similar to the observed increase in plasma levels of ANGPTL8 in individuals with obesity from our study cohort, plasma levels of hsCRP were also observed to be significantly higher in participants with obesity compared to those of normal-weight and overweight.

We observed significantly higher levels of chemerin in both the overweight and obese subgroups (see **Figure 2**). Additionally, we showed that higher chemerin levels were associated with an increased likelihood of obesity (see **Table 2**). We also reported a positive correlation between chemerin and ANGPTL8 levels (see **Figure 3**). Chemerin is an established adipokine produced mainly in adipocytes and liver and has a role in glucose homeostasis, inflammation, energy metabolism, angiogenesis, and adipogenesis (38, 39). It can display both pro- as well as anti-inflammatory actions depending on the biological system. Chemerin was shown to be involved in the pathogenesis of inflammatory and metabolic diseases in several organs including adipose tissue, lung, cardiovascular system, digestive tract, reproductive system, and skin. Its role in lipid metabolism is regulated *via* its action on its receptor CMKLR1 which is highly expressed in white adipose tissue (40). Several human studies reported a positive association between circulating chemerin levels in adults showing metabolic syndrome phenotypes (41, 42). Similarly in children, many recent reports demonstrated elevated levels of circulating chemerin in participants with overweight/obesity compared to normal-weight controls (42–46). These reports also suggest an association between higher chemerin levels and metabolic complications. Our findings are concordant with these previous reports.

Our study demonstrated significantly higher levels of leptin in both the overweight and obese subgroups (see **Figure 2**). Additionally, higher leptin levels were seen associated with an increased likelihood of obesity (see **Table 2**). A positive correlation between leptin and ANGPTL8 levels was seen (see **Figure 3**). Leptin is a peptide hormone secreted mainly by the subcutaneous white adipocytes, whose main roles are to regulate satiety and caloric intake (47). The focal action of leptin in hypothalamic neurons leads to decreased caloric intake and raises energetic expenditure in the long-term (46). Furthermore, leptin secretion is higher in subcutaneous than in visceral adipose tissue (48). Leptin is overexpressed at the gene level in the adipose tissue of individuals with obesity (49). Furthermore, strong positive associations exist between plasma leptin levels and body fat percentage (49). Our data is an agreement with previous publications (47, 50). Obese children having high leptin levels may face early onset of puberty.

The study demonstrated a reasonable predictive power for ANGPTL8 on adolescent obesity. The area under curve (AUC) at 0.703 (95% CI 0.648 - 0.759) indicated an acceptable accuracy in predicting obesity. A value of 122.8 pmol/l could be used as the optimal threshold of ANGPTL8 level to distinguish obese individuals from non-obese individuals. At this threshold, 67% of obese individuals could be picked as true positives and 64% of non-obese individuals could be picked as true negatives. Future studies might consider incorporation of other ANGPTLs to enhance the predictive power and the sensitivity/specificity.

Aside from inflammation, obesity is also characterized by dyslipidemia. Hence, it is worth pondering whether the observed increase in ANGPTL8 levels in overweight and obese children can impact lipid profile. Lipoprotein lipase (LPL) is the key enzyme that regulates lipoprotein metabolism and lipid content. Specifically, LPL is responsible for the hydrolysis of triglycerides (TGLs) into free fatty acids (FFAs). ANGPTLs, especially ANGPTL3, 4, and 8, are known to inhibit the enzymatic activity of LPL (17, 51–59). Animal studies using knockout mouse models illustrated how both ANGPTL8 and ANGPTL3 can play a key role in the metabolic transition between fasting and feeding states (59, 60). A recent article described elegantly the mechanism of action of ANGPTL8 in fasted and fed states and how ANGPTL3 and 4 are involved in such a regulation (17). Based on the findings from the above-mentioned studies, it could be suggested that the elevated ANGPTL8 levels in our cohort could interfere with the function of LPL resulting in an increase in TGLs. In fact, it has been shown that the overexpression of ANGPTL8 can lead to an increase in plasma TGL levels in an ANGPTL3-dependent manner (58). Clinical studies have presented a number of approaches to target ANGPTLs and indicated their potential therapeutic benefits especially for cardiovascular diseases (CVDs) and lipid disorders (61). In rodents, the use of ANGPTL8 antisense oligonucleotide showed positive effects on lipid metabolism and prevented hepatic insulin resistance (62). Moreover, ANGPTL3 is already being utilized as a target; the Food and Drug Administration (FDA) has recently approved Evinacumab, a monoclonal antibody against ANGPTL3, as a lipid-lowering agent for individuals with familial hypercholesterolemia (63). Further, ANGPTL8 antibodies or inhibitors that prevent its interactions with ANGPTL3 can disturb or prevent the ANGPTL3-8 complex formation, resulting in insufficient ANGPTL3-8 complexes (leading to reduced LPL inhibition) (64).

Strengths of our study include (a) a reasonably large sample size that is representative of the Kuwaiti adolescent population and the use of several statistical approaches to robustly show the association between ANGPTL8 and obesity; (b) none of the participants was diagnosed with any form of diabetes and this allows our study to be focused only on obesity-related features; and (c) an ANGPTL-based prognostic model with reasonable accuracy is presented. The main limitations of this study is (a) the lack of data on the lipid profiles of the participants which prevented us from evaluating the association between ANGPTL8 and lipoprotein levels in this population; (b) the lack of data on sex hormones which prevented us from examining the impact of sex hormone levels on the ANGPTL8 levels and obesity levels; and (c) the lack of a cohort of obese adolescents with insulin resistance which prevented us from examining levels of ANGPTL8 in the context of obese diabetes. Also, the cross-sectional design of the study did not allow us to establish causality. Therefore, further investigations are needed to establish the role of ANGPTL8 in the development of childhood obesity and the nature of its association with inflammatory and obesity markers.

In conclusion, we demonstrated that circulating ANGPTL8 levels were elevated in overweight and obese adolescents in the Arab population from Kuwait. ANGPTL8 showed a strong positive correlation with hsCRP, leptin, and chemerin which are well-known risk factors specifically for obesity, and broadly for metabolic syndrome. These findings highlight the importance of ANGPTL8 in adolescent obesity and metabolic diseases and confirm the potential use of ANGPTL8 as a powerful early marker for obesity and metabolic disorders.

## Data availability statement

The raw data supporting the conclusions of this article will be made available by the authors, without undue reservation.

## Ethics statement

The studies involving humans were approved by the Ethics Committee of the Ministry of Health, Kuwait (No: 2015/248), The Ethics Committee of the Health Sciences Centre, Kuwait University (No: DR/EC/2338), and the Ethical Review Committee of Dasman Diabetes Institute (RA2017-026). Parents of the participants signed the informed written consent form prior to the enrolment of adolescents in the study. Verbal assent of the study participants was also obtained. The studies were conducted in accordance with the local legislation and institutional requirements. Written informed consent for participation in this study was provided by the participants’ legal guardians/next of kin.

## Author contributions

MH: Writing – original draft, Data curation, Investigation. AC: Methodology, Software, Writing – review & editing. MF: Writing – review & editing, Conceptualization, Investigation, Supervision. AR: Investigation, Writing – review & editing, Data curation, Formal analysis, Funding acquisition. IK: Formal analysis, Writing – review & editing, Methodology. PC: Formal analysis, Methodology, Writing – review & editing. TA: Formal analysis, Writing – review & editing, Visualization. NE: Formal analysis, Writing – review & editing, Methodology. FM: Formal analysis, Writing – review & editing, Resources. TT: Writing – review & editing, Methodology, Software, Validation. JA: Conceptualization, Formal analysis, Writing – original draft.
